# Anti-Inflammatory, Antioxidant, and Other Health Effects of Dragon Fruit and Potential Delivery Systems for Its Bioactive Compounds

**DOI:** 10.3390/pharmaceutics15010159

**Published:** 2023-01-03

**Authors:** Daniela Franceschi Nishikito, Ana Claudia Abdalla Borges, Lucas Fornari Laurindo, Alda M. M. Bueno Otoboni, Rosa Direito, Ricardo de Alvares Goulart, Claudia C. T. Nicolau, Adriana M. R. Fiorini, Renata Vargas Sinatora, Sandra M. Barbalho

**Affiliations:** 1 School of Food and Technology of Marilia (FATEC), São Paulo 17500-000, Brazil; 2 Department of Biochemistry and Pharmacology, School of Medicine, University of Marília (UNIMAR), São Paulo 17525-902, Brazil; 3 Laboratory of Systems Integration Pharmacology, Clinical & Regulatory Science, Research Institute for Medicines (iMed.ULisboa), Faculdade de Farmácia, Universidade de Lisboa, Av. Prof. Gama Pinto, 1649-003 Lisbon, Portugal; 4 Postgraduate Program in Structural and Functional Interactions in Rehabilitation, University of Marília (UNIMAR), São Paulo 17525-902, Brazil

**Keywords:** dragon fruit, pitaya, *Hylocereus*, antioxidant, anti-inflammatory, health effects, delivery system

## Abstract

Dragon fruit (*Hylocereus* genus) has the potential for the prevention of diseases associated with inflammatory and oxidative processes. We aimed to comprehensively review dragon fruit health effects, economic importance, and possible use in delivery systems. Pubmed, Embase, and Google Scholar were searched, and PRISMA (Preferred Reporting Items for a Systematic Review and Meta-Analysis) guidelines were followed. Studies have shown that pitaya can exert several benefits in conditions such as diabetes, dyslipidemia, metabolic syndrome, cardiovascular diseases, and cancer due to the presence of bioactive compounds that may include vitamins, potassium, betacyanin, p-coumaric acid, vanillic acid, and gallic acid. Moreover, pitaya has the potential to be used in food and nutraceutical products as functional ingredients, natural colorants, ecologically correct and active packaging, edible films, preparation of photoprotective products, and additives. Besides the importance of dragon fruit as a source of bioactive compounds, the bioavailability is low. The development of delivery systems such as gold nanoparticles with these compounds can be an alternative to reach target tissues.

## 1. Introduction

Due to the profound changes in lifestyle resulting from modern life, there has been a progressive increase in non-communicable chronic diseases. Among them, cardiovascular diseases (CVD) are among the most deadly diseases in the world (32% of all global deaths) in western countries. Besides mortality, these conditions are associated with the leading cause of disabilities. Therefore, preventive measures are crucial to reduce the risk factors. Fruit and vegetables are essential compounds of a healthy diet, and their consumption in adequate amounts can diminish the risk of obesity, diabetes, CVD, and some types of cancer. Estimates from the World Health Organization (WHO) indicate that inadequate consumption of fruit and vegetables is among the top ten risk factors for the global burden of diseases worldwide [1,2,3,4].

Over the years, countless fruits have been associated with the prevention of non-communicable chronic diseases, and pitaya has also been considered [5,6].

The dragon fruit of pitaya is a rustic fruit belonging to the Cactaceae family, the genus *Hylocereus.* It is known as dragon fruit due to the presence of bright red skin with overlapping green fins covering the fruit. Other common names given to this fruit are pitahaya, dragon pearl fruit, night-blooming cereus, strawberry pear, and Cinderella plant. Depending on the species, its fruits may have different characteristics, such as shape, presence of thorns, skin, and pulp color, reflecting high genetic variability [5,6,7].

The health-promoting potential of pitaya fruit is due to the presence of bioactive compounds related to numerous benefits such as anti-diabetic, anti-inflammatory, antioxidant, anti-cancer, and antimicrobial. As a result of these beneficial actions, the consumption of this fruit has increased in different regions worldwide [7,8].

**Figure 1:**
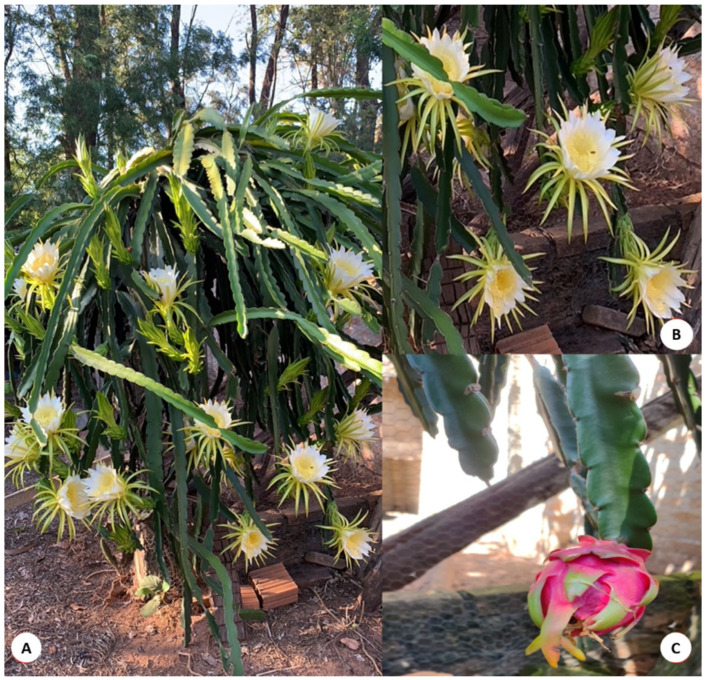
(**A**) pitaya plant, (**B**) flower, (**C**) fruit of *H. undatus*.

**Figure 2:**
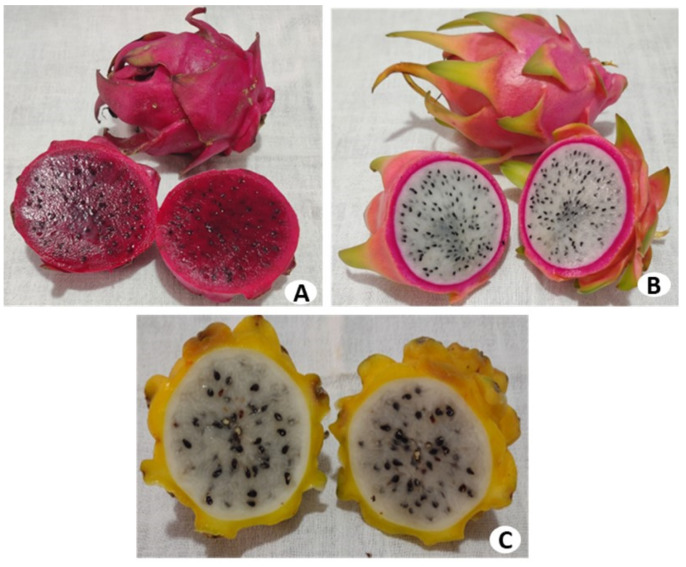
The fruit of *H. polyrhizus* (**A**), *H. undatus* (**B**), and *H. megalanthus* (**C**).

Dragon fruit is becoming popular in many countries and is consumed raw or can be added to drinks, jelly, and candies. In addition, the pigments can be used as a coloring agent in the pharmaceutical and food industries [9]. Three varieties that are distinguished by the color of the skin and flesh are mainly cultivated: *Hylocereus undatus* (possessing white flesh and red skin), *H. polyrhizus* (with red skin and red flesh), and *H. megalanthus* (yellow skin and white flesh) [6,10]. The fruit has an oval shape, and the pulp has a sweet and sour taste. The seeds are very small and black-colored [11,12]. Figure 1 and Figure 2 show the plant, the flower, and the fruit of *Hylocereus* spp.

In addition, there are some studies showing dragon fruit’s effects, but there is still a need for detailed scientific investigations to show the benefits this plant can bring to consumers. Moreover, dragon fruit can open a new window for developing multi-targeting drugs to prevent and treat several diseases and can be used for several technological applications in the food and pharmaceutical industries. For these reasons, we aimed to comprehensively review dragon fruit’s health effects, its economic importance, and its possible use in delivery systems. To the best of our knowledge, this is the first review considering the species *H. undatus, H polyrhizus*, and *H. megalanthus* and their role in health and technological applications.

## 2. Materials and Methods

### 2.1. Focused Question

The focal question for this review was: what are the effects of the genus *Hylocereus* fruit on health?

### 2.2. Language

We used only studies published in English, Spanish and Portuguese language.

### 2.3. Databases

For this review, we consulted the following databases: PubMed, EMBASE, Google Scholar, and COCHRANE. 

### 2.4. Study Selection

In this review, we included Randomized Controlled Trials (RCTs) that studied the effects of the genus *Hylocereus* on human health. Moreover, in vitro and in vivo studies were considered. The mesh terms applied were pitaya or dragon fruit or *Hylocereus* or *H. undatus* or *H. polyrhizus* or *H. megalanthus* and health or glycemia or blood pressure or cholesterol or lipids or body mass index or obesity or body weight or cardiovascular diseases. The inclusion criteria were RCTs, placebo-controlled studies, quasi-experimental studies, and in vivo and in vitro models. Only full texts were added. The exclusion criteria were articles performed in languages other than English, Spanish, and Portuguese, poster presentations, editorials, and case reports. Review texts helped in the discussion section.

### 2.5. Data Extraction

**Figure 3:**
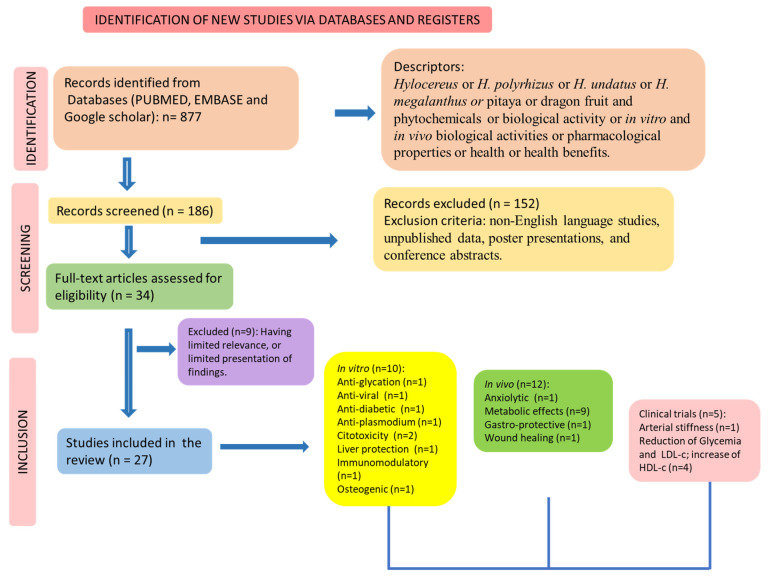
Flow diagram showing the search of the included studies performed with humans (according to Page et al. [13]).

We did not restrict a period for the search for trials. The search for human, animal, and in vitro studies followed Preferred Reporting Items for a Systematic Review and Meta-Analysis (PRISMA) guidelines [13] (Figure 3).

### 2.6. Quality Assessment

To perform a quality assessment, the evaluation of the presence of risk of bias in the RCTs (detection, selection of the study, and reporting biases) was performed according to *The Cochrane Handbook for Systematic Reviews of Interventions* [14]. Additionally, the evaluation of the presence of risk of bias in the included animal studies (detection, selection of the study, and reporting biases) was performed according to the Systematic Review Centre for Laboratory animal Experimentation RoB tool (SYRCLE’S guidelines) [15].

### 2.7. Evaluation of Economic Importance and Technological Applications

Other studies, such as research studies, reviews, and metanalysis, were evaluated to help describe the possible technological applications of pitaya and the economic importance of this plant.

## 3. Results

Many studies have shown that dragon fruit can benefit numerous health problems and work as an analgesic, antioxidant, anti-diabetic, anti-cancer, cardio-protective, liver protective, and neuroprotective. However, only five clinical trials were found and included. All of them were performed with *H. polyrhizus.* Three of them were performed in Malaysia [16,17,18], one was performed in Indonesia [19], and one in the United Kingdom [20]. The primary outcomes were improved glycemia, lipid profile, and antioxidant status [16,17,18], flow-mediated dilation, and arterial stiffness [20]. In vitro studies showed anti-glycation, anti-diabetes, anti-viral, anti-plasmodium, hepatoprotective, immunomodulatory, and osteogenic effects. In vivo studies have also shown the effects of the genus *Hylocereus* on animal models (Table 1). Lira et al. [21] demonstrated the anxiolytic effects of pitaya in zebrafish. Anand Swarup et al. [22] demonstrated that pitaya’s pulp extracts have potent actions against arterial stiffness, reducing blood pressure and controlling pulse wave velocities. Holanda et al. [23] investigated the effects of *Hylocereus* on the lipid and glycemic profile of rats. Silva et al. [24] investigated the use of red pitaya in glycemia. Yeh et al. [25] and Ramli et al. [26] showed that pitaya affects the liver’s health, reducing liver lipids content and enhancing the enzymatic potential of the hepatocytes against liver injuries. Ramli et al. [27] and Song et al. [28] demonstrated more profoundly the metabolic effects of pitaya, principally in enhancing lipid metabolism, diminishing obesity, and augmenting the gut microbiota content of *Akkermansia*. Macias-Ceja et al. [29] demonstrated that pitaya could exert anti-inflammatory actions in rats’ gastrointestinal systems, principally regulating pro-inflammatory pathways and diminishing the chance of the rats developing colitis. Lastly, Perez et al. [30] demonstrated that many extracts of pitaya could exert wound-healing effects against diabetic-induced wounds principally by enhancing tensile strength, hydroxyproline, DNA, total proteins, collagen content, and epithelization. The evaluation of the bias of the animal studies is shown in Table 2.

## 4. Discussion

### 4.1. Bioactive Compounds of Hylocereus Species

The phytochemical compounds found in dragon fruit mainly belong to phenols, flavonoids, sterols, fatty acids, and tocopherol. Among the numerous bioactive compounds found in the pulp and peel, it is possible to highlight the presence of ascorbic acid, tocopherol, thiamin, niacin, and riboflavin, minerals like calcium, magnesium, potassium, phosphorous, betacyanin, β-carotene, lycopene, p-coumaric acid, protocatechuic acid, vanillic acid, gallic acid, syringic acid, and p-hydroxybenzoic acid. The properties of these phytochemicals are shown in Table 3 [6,33,34,35,36]. The analysis performed on *H. undatus* pulp with different solvents indicated the presence of carbohydrates, tannins, saponins, anthocyanins, quinones, glycosides, terpenoids, triterpenoids, phenols, acids, and steroids in aqueous extract. In the methanolic extract, carbohydrates, tannins, saponins, flavonoids, alkaloids, anthocyanins, cardiac glycosides, terpenoids, triterpenoids, phenols, acids, and steroids were identified. Extraction with hexane showed carbohydrates, saponins, anthocyanin, quinones, phenols, and acids. Carbohydrates, saponins, alkaloids, cardiac glycosides, triterpenoids, phenols, and coumarins were extracted in chloroform, as well as carbohydrates, tannins, saponins, flavonoids, alkaloids, anthocyanin, cardiac glycosides, terpenoid acids, triterpenoids and steroids in ethyl acetate [37].

Al-Mekhlafi et al. [38] evaluated the differences between the varieties and origins of pitaya extracted with methanol-water and methanol based on proton nuclear magnetic resonance. The results showed a high concentration of phenolic compounds in the red fruit in Israel and Thailand; however, higher levels of glucose and fructose were observed in the white and yellow pulped fruits from Israel. A study by Arivalagan et al. [39] also showed that white pitayas have higher yields and higher levels of these sugars, while fruits with red pulp indicated antioxidant potential and high concentrations of phenolic compounds. Other sugars identified by the authors were mannose, arabinose, inositol, ribose, xylose, and sucrose.

Studies carried out on *H. polyrhizus, H. megalanthus,* and *H. undatus* indicated the presence of polysaccharides, flavonoids, phenol, and betacyanins [40,41]. The pitaya peel contains high levels of betacyanin, an antioxidant substance with antimicrobial activity, and the presence of pigments that indicate its possible use as a preservative and/or food coloring with benefits for the health of the consumer. There are seven betacyanins described in red pitaya (betanin, isophyllocactin, betanidin, isobetanidin, bougainvillein-R-I, isobetanin, and lhyllocactin) [42]. According to Choo et al. [43], who evaluated the bioaccessibility of betacyanins in fermented beverages and red dragon fruit juice, it was possible to verify that the bioactive compounds suffered great losses in environments that simulate intestinal digestion. It was also observed that the fermented beverage presented greater antioxidant capacity than the juice.

Ariffin et al. [44], when separating the seeds, extracting the oil, and evaluating two varieties of pitaya (*H. undatus* and *H. polyrhizus*), found a significant percentage of essential unsaturated fatty acids (linoleic acid and linolenic acid, and two isomers of oleic acid). According to the authors, although pitaya seed oil contributes to the fatty acid content, the seed/fruit ratio is relatively low.

**Table 3:** Main bioactive compounds of pulp, peel, and seeds of the genus *Hylocereus*.

References	Bioactive Compounds	Pulp, Peel, or Seed	Molecular Structures	Health Effects	Concentration (Dry Weight)
[45,46]	Anthocyanins (cyanidin 3-glucoside, delphinidin 3-glucoside, and pelargonidin 3-glucoside)	Peel and pulp	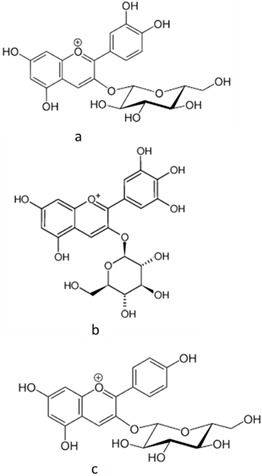	Anti-inflammatory, antioxidant	0.82 ± 0.17 a 12.67 ± 0.63 mg/100 g (pulp) and 44.3865 ± 1.3125 mg/100 g (peel)
[6,47,48,49]	Ascorbic acid	Peel and pulp	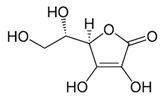	Antioxidant, anti-cancer, anti-diabetic, anti-lipidemic	2.94 ± 1.1 a 5.64 ± 0.7 mg/100 g (pulp) and 175 ± 15.7 µmol Trolox equivalents antioxidant capacity (TEAC)/g (peel)
[49,50]	Betacyanin	Peel and pulp	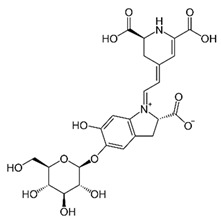	Anti-microbial, anti-viral, pigments, hypolipidemic	10.3 ± 0.22 a 82.79 ± 0.55 (pulp) and 13.8 ± 0.85 a 18.67 ± 0.50 mg/100 g (peel)
[51,52]	Lycopene	Pulp	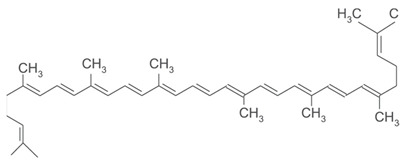	Antioxidant, anti-diabetic, anti-cancer	3.2 ± 0.6 µg/100 g (pulp)
[6,52,53]	β-carotene	Pulp	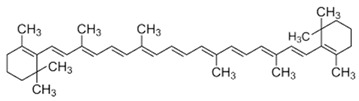	Antioxidant, anti-diabetic, anti-cardiovascular diseases	209.1 ± 0.1 µg/100 g (pulp)
[10,54]	Tocopherol	Seed	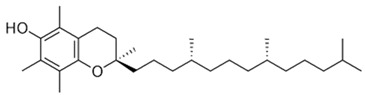	Antioxidant, anti-cancer, anti-diabetes, anti-cardiovascular diseases	150 µg/100 g (seed)
[6,55]	p-Coumaric acid	Pulp and seed	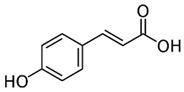	Antioxidant, anti-diabetic, anti-lipidemic	0.365 a 1.52 ± 1,4 mg/100 g (seed) and 0.16 mg 100 g (pulp)
[10,49,56,57]	Gallic acid	Peel, pulp, and seed	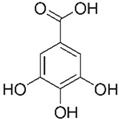	Antioxidant, anti-diabetic, anti-obesity	24.0 ± 2.7 a 53.2 ± 6.2 mg GAE/100 g (pulp), 39.9 mg GAE/100 g (peel)and 375.1 mg GAE/100 g (seed)
[6,58]	Syringic acid	Peel, pulp, and seed	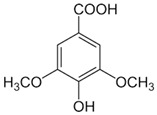	Antioxidant, anti-diabetes, anti-cardiovascular diseases, anti-cancer	Variando de 0.095 a 65.10 ± 0.04 µg/100 g (peel and pulp) Seed (NA)
[5,49,59]	Vanillic acid	Peel and pulp	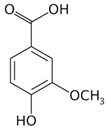	Antioxidant, anti-inflammatory, anti-diabetic, anti-proliferative, and anti-atherogenic activities	0.64 mg/100 g (peel and pulp)
[6]	Phthalic acid	Peel	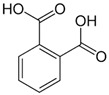	Antioxidant	(NA)
[39]	Benzoic acids derivatives: salicylic acid (a), 3- hydroxyl benzoic acid (b), 4-hydroxy benzoic acid (c).	Peel and Pulp	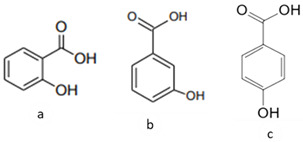	Antioxidant	Variando de 0.48 ± 0.0 a 11.97 ± 0.8 µg/100 g (pulp and peel)
[6,49,60]	Caffeic acid	Peel and seed	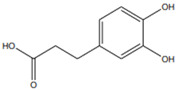	Antioxidant	0.08 mg/100 g (pulp and peel)
[45,61]	Quercetin	Peel and pulp	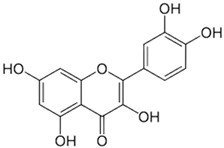	Antioxidant, anti-inflammatory, anti-diabetic	3.43 mg/100 g (pulp) Peel (NA)
[44,48,62,63,64]	Fatty acids: oleic acid (a), linoleic acid (b), linolenic acid (c), and palmitic acid (d)	Pulp and seeds	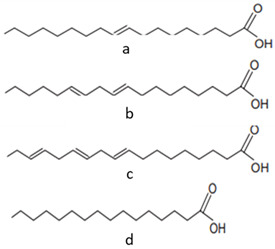	Nutraceutical activity	129.11 (a), 252.65 (b), 5.33 (c), 62.74 (d) mg/100 g (pulp) and 60 (a), 21 (c), 13 (d) g/100 g (seeds)

NA: Not Available

### 4.2. Healthy Benefits of Hylocereus Species

Dragon fruit has been used as a medicinal food since ancient times by Mayas—using the fruit and the flowers as a hypoglycemic, wound disinfectant and diuretic, for dysentery, tumor dissolution, and as a healing agent. Moreover, the flowers and the seeds can be used as beverages for gastritis, as laxatives, and to improve kidney function. These properties are derived from the multi-bioactive compounds found in this plant, mainly in the fruit. Furthermore, it shows no toxicity since a study with dragon fruit extract at a dose of 1250 to 5000 mg/kg did not produce interferences or abnormalities in the organs of animal models [65,66,67,68]. Below it is possible to find some effects of pitaya fruit. 

As the results found in Table 1 show, the use of pitaya pulp or its bioactive compounds can exert anxiolytic, antioxidant, and anti-inflammatory effects in animal models. Improvements in hepatic steatosis and reduction of liver injury are also observed, in addition to balancing the intestinal microbiota. These results in animal studies allow us to say they can also be observed in humans.

#### 4.2.1. Antioxidant Effects by Species

*Hylocereus. polyrhizus* is rich in betalains and other bioactive compounds such as vitamins and phenolic compounds that exert relevant antioxidant properties and, for these reasons, are related to the prevention of several human diseases. The oil results from the seeds, and the peel is also an essential source of antioxidant compounds. The peel of *H. undatus* possesses more flavonoids than the flesh [65,66,67,69] (Table 3).

The antioxidant properties of dragon fruit extract were investigated. Total antioxidant status was reduced in pre-diabetic and normocholesterolemic subjects that consumed red pitaya [16,18]. Harahap and Amelia [70] showed that fruit extract could minimize oxidative damage production in animals submitted to physical exercises. Other authors have shown that the pulp extract can decrease oxidative damage in STZ-induced diabetes in rats [71].

**Figure 4:**
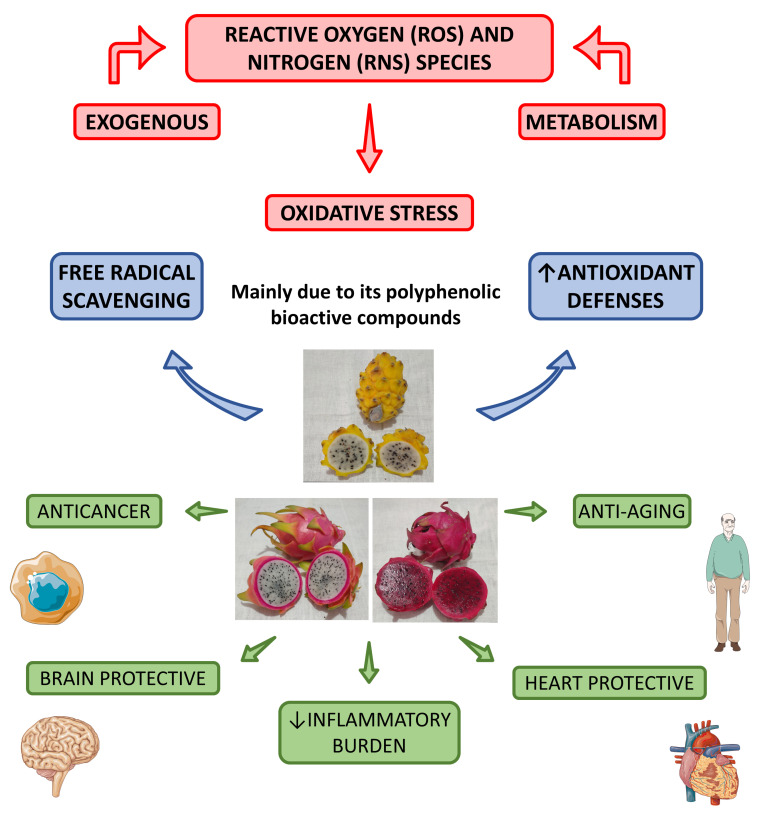
Main antioxidant effects of *Hylocereus* species and their health effects. ↑—increase; ↓— decrease.

Putri et al. [72] showed that the intake of red dragon fruit could reduce malondialdehyde levels in diabetic rats and, for these reasons, is associated with reducing the oxidative stress related to this disease. Figure 4 shows the main antioxidant effects associated with the *Hylocereus* species.

#### 4.2.2. Anti-Inflammatory Effects

Besides the antioxidant actions, dragon fruit can also exert anti-inflammatory actions. Saenjum et al. [45] found anthocyanins (cyanidin 3-glucoside, delphinidin 3-glucoside, and pelargonidin 3-glucoside) in the pulp and peel of pitaya red. The authors found that the pulp enriched with the first anthocyanin (cyanidin 3-glucoside) inhibited the synthesis of reactive oxygen and nitrogen species, cyclooxygenase-2 (COX-2), and inducible nitric oxide synthase (iNOS), in in vitro models and without resulting in cytotoxicity.

Another study showed that the dragon flesh and peel extract and the isolated squalene led to the inhibition of pro-inflammatory enzymes such as cyclooxygenase-2 lipoxygenase and acetylcholinesterase and concluded that this fruit could produce a significant potential for the control and management of inflammatory processes through different pathways that may include, prostaglandin, leukotriene, and cholinergic pathways [73].

Amim et al. [74] found different effects (anti-inflammatory, antioxidant and anti-bacterial actions) in the extracts of *H. polyrhizus* and *H. undatus* (the aqueous extract of *H. polyrhizus* showed most significant effects than *H. undatus*). The authors found that the aqueous extract of these species resulted in more protective anti-inflammatory and antioxidant activities compared to the ethyl acetate and ethanolic extracts.

Al-Radadi et al. [75] found significant anti-inflammatory, anti-diabetic, anti-Alzheimer, and cytotoxic effects of gold nanoparticles using *H. polyrhizus*. 

**Figure 5:**
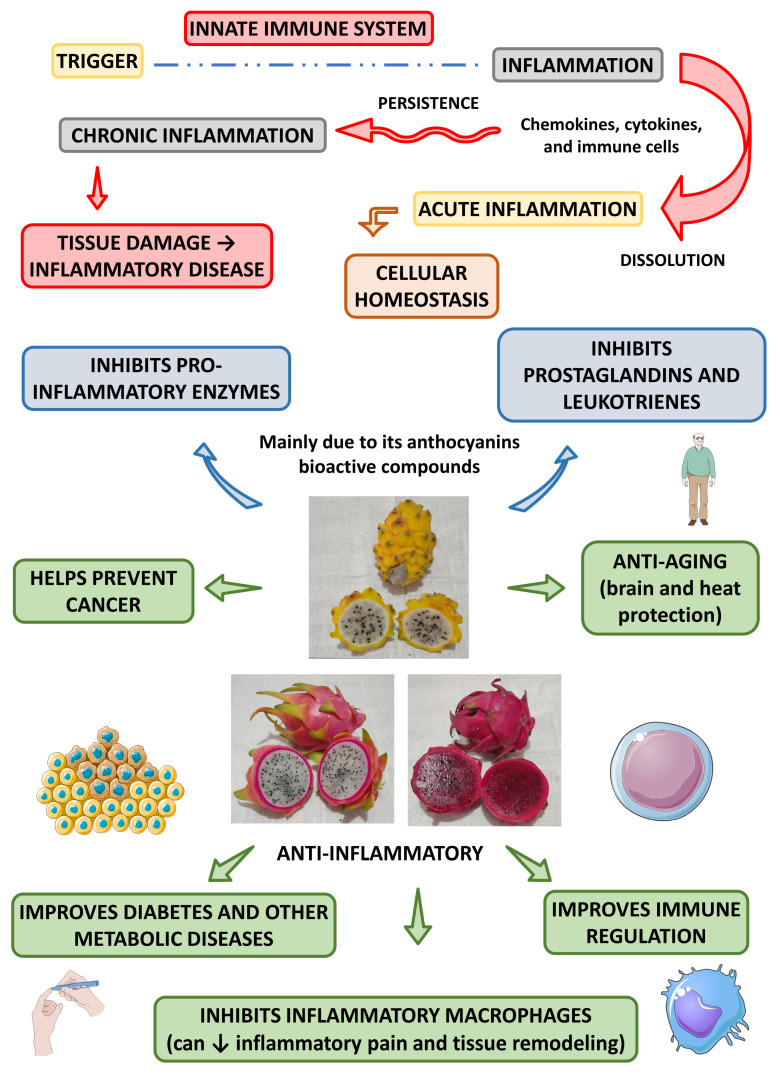
Main anti-inflammatory effects of *Hylocereus* species and their health effects. ↓—decrease.

Figure 5 shows the most prominent anti-inflammatory effects associated with the *Hylocereus* species.

#### 4.2.3. Prebiotic Effects

The main carbohydrates in white and red flesh dragon fruit are glucose, fructose, and oligosaccharides. The mixed oligosaccharides are resistant to hydrolysis by human α-amylase and artificial human gastric juice (about 34.88% and 4.04%, respectively). Moreover, the mixed oligosaccharides can also stimulate the growth of lactobacilli and bifidobacterial, thus showing prebiotic properties [76].

Dasaesamoh et al. [77] showed that the fecal fermentation of pitaya oligosaccharides increased the populations of Bifidobacteria and Lactobacillus and reduced the populations of Bacteroides and *Clostridium*. Moreover, this fecal fermentation resulted in a positive prebiotic effect. Lactic acid, acetic acid, propionic, and butyric acids were produced at substantial concentrations.

#### 4.2.4. Antimicrobial Effects

In a study to investigate the antimicrobial effect of red pitaya peels, Temak et al. [78] found that the extract has efficient in vivo and in vitro effects against several microorganisms, such as *Escherichi coli* and *P. aeruginosa.*

Sushmitha et al. [79] investigated the effects of *H. undatus* seeds in Gram-negative and Gram-positive bacterial species and found that the minimum inhibitory concentration is 50 µL for *Staphylococcus aureus* and *Escherichia coli*. Tenore et al. [80] also found antimicrobial activity for hexane, chloroform, and ethanol extract of the skin of *H. undatus* and showed inhibition of the growth of Gram-negative and Gram-positive bacteria.

Extract of the peel and fruit of the red dragon fruit can also exert anti-fungal actions against *Rhizoctonia solani, Candida albicans, Aspergillus flavus, Botrytis cinerea, Fusarium oxysporum*, and *Cladosporium herbarum* [80].

#### 4.2.5. Anti-Cancer Effects

Some studies have shown the anti-cancer potential of dragon fruit. Divakaran et al. [81] aimed to evaluate the ability of this fruit to produce nanoparticles and found they can significantly inhibit the growth of MCF-7 breast cancer cells. 

Another study showed that the fecal fermentation of pitaya oligosaccharides augmented the populations of Lactobacillus and decreased the populations of Bacteroides and Clostridium, and resulted in the production of lactic acid, acetic acid, propionic and butyric acids that can inhibit Caco-2 cells and has a potential for risk reduction in colon cancer [77].

In a very interesting study, Padmavathy et al. [37] demonstrated that methanol extracts of *H. undatus* have promising anti-cancer effects against human liver cancer (HepG-2) cells.

Wu et al. [82] investigated the anti-proliferative effect of red pitaya on B16F10 melanoma cells. They showed that the peel has stronger inhibition of the growth of these cancer cells than the flesh. The authors concluded that both peel and flesh are rich in polyphenols and good sources of antioxidants, and for these reasons, the peel can inhibit the growth of melanoma cells.

The anti-cancer activities promoted by pitaya are related to several bioactive compounds such as phenolic acids, flavonoids, and betacyanin [83,84].

**Figure 6:**
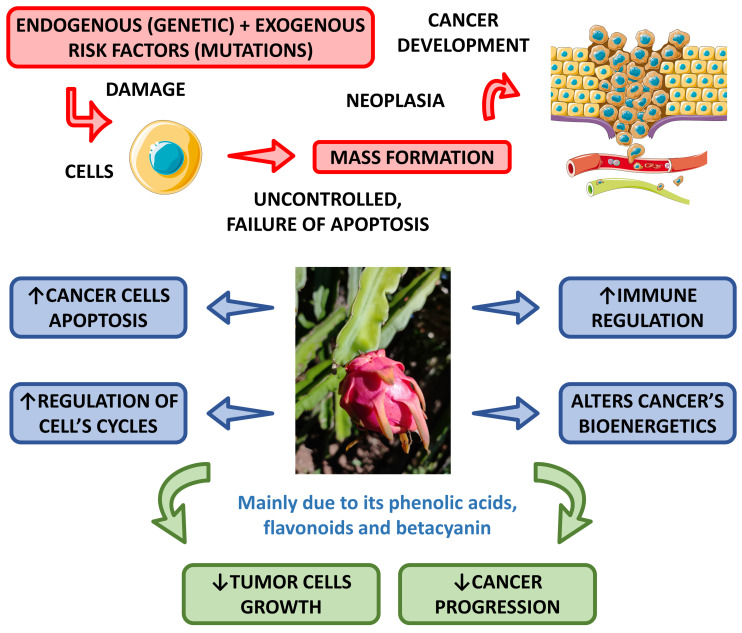
Main anti-cancer effects of *Hylocereus* species. ↑—increase; ↓—decrease.

Figure 6 shows the most pronounced anti-cancer effects associated with the *Hylocereus* species.

#### 4.2.6. Anti-Diabetic Effects

Many studies have demonstrated that the consumption of red pitaya can reduce glycemia in humans [16,17,18]. In a systematic review and meta-analysis, Poolsup et al. [8] found that dragon fruit can be used to prevent diabetes.

The study of Putri et al. [72] showed that pitaya associated with metformin could significantly decrease glycemia and HOMA-IR (homeostasis model assessment-Insulin Resistance) in type 2 diabetic rats. The authors suggested that red dragon fruit could be used as an alternative to metformin due to its effectiveness in decreasing HOMA-IR (and thus, insulin resistance) and malondialdehyde levels. Moreover, the consumption of red pitaya promoted a hypoglycemic effect in dyslipidemic C57BL/6 mice, contributing to reducing the risk of insulin resistance [85].

Fadlilah and Sucipto [19] found that pitaya (*H. polyrhizus*) effectively reduces fasting blood sugar levels in students who consume high calories daily.

Marietta et al. [86] investigated the effects of red pitaya skin extract on glycemia and lipid profile of diabetic and dyslipidemic male Wistar rats and found no significant reduction in glycemia.

#### 4.2.7. Anti-Lipidemic Effects

The use of red pitaya can improve lipid profile, decrease total cholesterol, LDL-c, and triglycerides, and increase HDL-c levels in normocholesterolemic subjects, pre-diabetic, and type 2 diabetic patients [16,17,18]. 

The consumption of red pitaya also showed benefits in lipid levels in dyslipidemic C57BL/6 mice, contributing to reducing cardiovascular diseases [85].

The study of Marietta et al. [86] evaluated the effects of the consumption of red pitaya skin extract on the lipid profile of male Wistar rats with diabetes and dyslipidemia and did not find a significant reduction in the lipid profile of these animals.

Setiawan et al. [87] investigated the effects of *H. polyrhizus* peel powder on male Balb-c mice. Their results showed decreased triglycerides, total cholesterol levels, and LDL-c. Moreover, they observed an increase in HDL-c levels. The authors concluded that the use of the peel of this fruit could improve the blood lipid of mice with hyperlipidemia.

**Figure 7:**
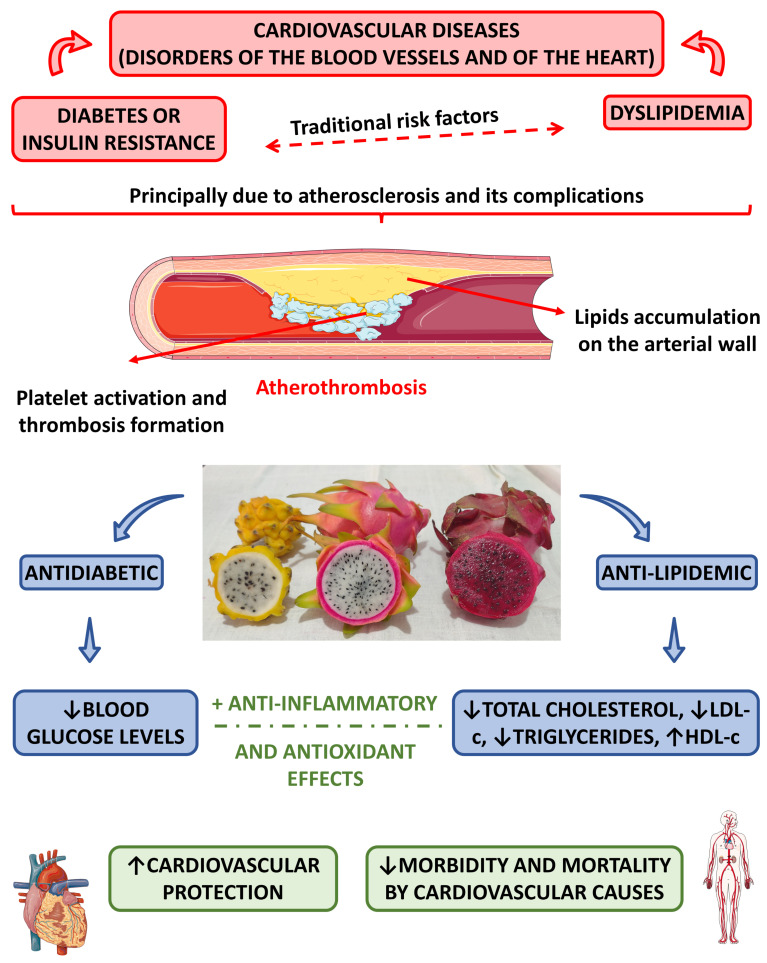
Cardiovascular protective effects of the *Hylocereus* species. ↑—increase; ↓—decrease; HDL-c—high-density lipoprotein cholesterol; LDL-c—low-density lipoprotein cholesterol.

Figure 7 shows the most relevant anti-lipidemic and anti-diabetic effects associated with the *Hylocereus* species and how these effects are associated with atherosclerosis. Table 4 summarizes some in vitro studies performed with pitaya.

#### 4.2.8. The Effects of Dragon Fruit on Humans: Evaluation of Clinical Trials

Based on the inclusion and exclusion criteria for the search of studies that were performed using pitaya in humans, only five clinical trials were added to Table 5. The risk of bias observed in these studies is found in Table 6. These trials are commented on below.

Shafie et al. [18] investigated the effects of using spray pitaya powder (SPP) in normocholesterolemic subjects. All the doses led to reductions in total cholesterol and LDL-c (*p* < 0.05); however, the higher the dose, the higher the reduction in cholesterol levels. For the blood levels of triglycerides and HDL-c, there were significant differences (decrease and increase, respectively) in the higher dose (*p* < 0.05). 

In the study of Akhiruddin [16], the pre-diabetic participants were allocated to receive pitaya powder (obtained from *H. polyrhizus*) in different concentrations. At the end of the treatment, compared to the control group, the group that received 100 g per day showed the best reduction in blood glucose (22.90%, *p* < 0.05). The same was observed for the lipid levels; there was a significant reduction (*p* < 0.05) in total cholesterol (26.44%), triglycerides (20.54%), and LDL-c (69.55%) (the higher the dose, the higher the percentage of reduction). Moreover, the group that received 100 g of the red pitaya powder presented an increase of 63.8% in HDL-c levels. Although the results of these two studies are very interesting, the authors did not mention the participants’ age and gender. 

Abd Hadi et al. [17] investigated the effects of consuming pitaya fruit in type 2 diabetic subjects that receive red pitaya daily. Participants were subjected to a seven-week trial consisting of phase 1 (one week) and phase 2 (4 weeks) of treatment, and phase 3 (2 weeks of wash-out). The results showed a reduction (*p* < 0.05) in glycemia and lipids.

Fadlilah and Sucipto [19] investigated the effects of red pitaya intake on students’ blood pressure and glycemia after excessive food consumption. The obtention of the samples was performed using consecutive sampling techniques with thirty-two respondents each for the treatment and control groups. The glycemia pre-test and post-test in the treated group were 87.5 mg/dL and 81.0 mg/dL, respectively, and in the control group were 83.00 mg/d and 82.0 mg/dL, respectively. The systolic blood pressure pre- and post-test were 112 mmHg and 115 mmHg, respectively, in the control group, compared to 117 mmHg and 109 mmHg, in the intervention group. The diastolic blood pressure pre-test and post-test were 79 mmHg and 82 mmHg in the control group; in the treated group, it was 77 mmHg and 70 mmHg in the pre- and post-test, respectively.

The study performed by Cheok et al. [20] showed that pitaya intake significantly improved acute low-mediated dilation (*p* < 0.001) compared to placebo. Pulse wave velocity was significantly decreased (*p* = 0.003), whereas the augmentation index improved after fourteen days (*p* = 0.02). Although the results of this trial are interesting, it is biased due to the small sample and a short-time follow-up that could interfere with the interpretation of the results.

Since the pulp and the skin of *Hylocereus* can exert antioxidant and anti-inflammatory actions, the consumption of this plant, as shown by the trials mentioned above, can reduce the risk of several diseases related to oxidative stress and inflammatory processes. These diseases include diabetes, dyslipidemia, metabolic syndrome, and cardiovascular conditions.

As we can observe in the results of studies on humans, the use of dragon fruit can prevent or improve numerous risk factors for developing metabolic syndrome and cardiovascular complications, which are among the leading causes of death today. This can be observed in the reduction of glycemia, total cholesterol, LDL-c, improvement in blood pressure and total antioxidant status, and an increase in HDL-c levels.

### 4.3. Technological Applications and Economic Importance

With the opening of trade, the world fruit market has become more competitive and open to novelties, such as native and exotic fruits, mainly due to media disclosures about the benefits of fruit consumption, highlighting them as healthy, balanced, functional and diversified—with their colors, shapes, smell, and flavors, which aroused the desire for native and exotic fruits. The pitayas are within the exotic fruits group and have aroused consumers’ interest. Due to their high commercial and tolerance to water stress, they also aroused the interest of fruit growers in their planting and cultivation [96,97,98,99] 

In the exotic fruit market, pitaya has stood out in Brazil and other parts of the world since it has agronomic and economic potential due to its rusticity. Commercial value is high since production, transport, and distribution costs require specialized logistics and quality control [100,101,102] 

Each part of dragon fruit (pulp, rind, seeds, flower buds, dried flowers) has tremendous nutritional value in terms of antioxidants, fiber, vitamin C, and minerals, especially calcium and phosphorus, nutritional attributes that caught researchers’ attention so that it can be used and processed in different products. The fruit peel has the potential as an anti-bacterial agent, natural dye, and antioxidant, in addition to the nutritional benefits of ripe fruit. The young stem and fresh flower buds are also edible and can be used as a vegetable. The dried flowers were considered to make a tea rich in antioxidants. The fruit pulp can be used to produce juice, wine, jam, and other products. The skin is used for the extraction of natural food coloring as well as a source of pectin, and the seeds are mainly used to extract the oil, which contains about 50% essential fatty acids. In addition, seeds are an ingredient in many food products, such as syrups, ice creams, candies, and yogurts [5,103]. 

Pitaya skins are rich in betacyanins, which contribute greatly to the fruit’s abundant red appearance. The main betacyanin compounds are betanin, isobetanine, phyllocatin, isophyllocatin and hylocerenin. Due to the numerous bioactive compounds that can be recovered, the use of food products as functional ingredients or natural colorings would help improve the overall added value of pitaya and reduce the environmental impact. Unlike anthocyanins, betacyanins can maintain desirable pH stability in the range of 3 to 7, and betacyanins can have stronger antioxidant activities, which suggests great potential to be applied in the food and nutraceutical industries. [94,103,104]. Betacyanins, in particular, cover a class of natural red-violet coloring pigments with chemical and biological properties for broad-ranging applications in the food, cosmetic, and pharmaceutical industries [50,105]. A study by Belluci et al. [106] found that red pitaya extract can be considered a natural additive to improve the color and sensory acceptance of meat products.

Betacyanins can be applied as a natural dye to improve the color property. On the other hand, the dietary fiber in dragon fruit peel can be used to replace partial fat in products such as ice cream. In one study, *H. polyrhizus* peel was introduced as a fat substitute to formulate high-fiber, low-calorie ice cream [107,108]. 

Another use of dragon fruit in the industry is the development of environmentally friendly, active packaging and edible films to extend the shelf life of food products, with the recovery of betacyanins and other phenolic compounds [109,110]. Betacyanins can be recovered to be used as active packaging or as an edible coating [109,111].

Tamagno et al. [112] suggest using microencapsulated pitaya (*H. undatus*) pulp extract as a food supplement to minimize the effects of copper poisoning and to avoid oxidative damage caused by metals.

According to Hübner [113], adding dragon fruit pulp and ginger in the secondary fermentation process produced beers with superior antioxidant capacity compared to the control beer, suggesting an alternative flavor, aroma, and antioxidant compounds in this style of beer. 

The results of a study performed by Vijayakumar et al. [114] demonstrated that *H. polyrhizus* bark extract is a potent antioxidant with photoprotective properties. In addition, the phenolic compounds and flavonoids helped the overall antioxidant properties, the high sun protection factor value, and broad-spectrum UVA and UVB photoprotection.

Moreover, a study using red pitaya extract showed that it presents substantial blood biocompatibility and physiological stability. The green production of gold nanoparticles with fruit extract can be an alternative to chemical production and is important for biological and medical applications [75]. 

### 4.4. Delivery Systems for Hylocereus Compounds

With the increased incidence of chronic degenerative diseases, consumers are increasingly interested in nutritional, healthy, and safe food. For these reasons, the demand for natural products has increased substantially. For example, the search for pigments to substitute artificial colorants or additives in food products has been a desired target. Moreover, natural compounds may also be associated with anti-inflammatory and antioxidant actions that would meet the need for prevention and, or, as adjuvants in treating many chronic conditions [115,116,117,118,119,120]. On the other hand, bioactive compounds may not have the necessary bioavailability to reach target tissues in the body and rapidly undergo degradation under many types of storage, processing, or the gastrointestinal environment, due to oxygen, temperature, pH, light, and enzyme exposure. The possible effectiveness of bioactive compounds depends on their bioavailability, and, for these reasons, this is an obstacle to overcome [121,122]. 

The need to study drug delivery systems aims to maximize therapeutic efficacy or minimize side effects by impacting the absorption, distribution, metabolism, and elimination of a drug compound [123]. The elaboration of an edible delivery system for active bio compounds is challenging due to the differences in solubility, bioavailability, and potential for novel food and pharmaceutical applications. One of the challenges is that all materials involved must have quality control to be used in food and sufficient chemical and physical stability to withstand the conditions to which they will be subjected (such as changes in temperature, solubility, pH, etc.) [43,124,125].

Betacyanins in the *Hylocereus* species are natural water-soluble nitrogenous pigments and red-violet. Together with yellow betaxanthins, they constitute betalains. However, as with other bioactive compounds, betacyanins exhibit a short shelf-life and low bioavailability (some studies show that betanin bioavailability is lower than 1% of the ingested amount). Betanin bioavailability was found to be around 0.5–0.9% among human volunteers [126]. In another study, the authors demonstrated that only 0.2 lM plasma concentration is retained after ingestion of 500 g of the pulp of cactus pear fruit, resulting in 16 mg of betanin and a bioavailability near 0.68% [127]. Choo et al. [43] showed that betacyanins bioaccessibility in fermented red dragon fruit drink and pressed red dragon fruit juice undergoes reduced loss (around 25%) in the gastric-like environment and higher loss during intestinal digestion. After intestinal digestion, fermented red dragon fruit drink retained around 46.0% of betanin and the fruit juice retained approximately 44.0% (concentration of betanin of 17.1 mM and 12.4 mM, respectively). The findings of this study suggested that betacyanins are bioaccessible; however, fermentation can augment bioavailability. A study performed by Sawick et al. [128] revealed that betacyanins suffered intensive degradation when fermented juice prepared with red beet was administered via gavage to rats and absorbed from the stomach. Nineteen betacyanins (eight were native compounds, and 11 were metabolites) were found in the physiological fluids of the rats. Peak amounts were found in the portal vein 15 and 30 s after administration of the extract (respectively, 0.8 and 12.3 µM for 5 mg and 20 mg betacyanins). Elimination (excretion) of these compounds in urine peaked after 15 and 30 s (0.1 and 3.3 µmol/h after 5 and 20 mg), respectively. In another animal study, Krant et al. [129] showed that betanin underwent metabolization mainly in the stomach wall (74%), followed by the colon (60%), and in a small percentage, in the small intestine (35%). Only a tiny percentage underwent liver metabolization, and about 2.7% was eliminated in urine and feces [75]. Furthermore, the presence of these compounds is influenced by external factors, such as food source, food matrix (type of product, structure, composition, processing, and viscosity), processing (pH, viscosity, fermentation, and Brix), water activity, temperature, oxygen, light, pH, mechanisms of absorption, and catalytic enzymes [126,127,130,131,132,133,134,135].

The preparation of nanoliposome phospholipids augmented the stability of betanin and resulted in higher 2,2-diphenyl-1-picrylhydrazyl radical-scavenging capacity in gummy candies than those containing free betanin [132]. Encapsulated betalain (w/o/w double emulsions) showed higher encapsulation efficiency and emulsion stability [134]. In the hydrophilic form, betacyanin from red dragon fruit extract is a high-value bioactive component with a plethora of applications as functional food or nutraceuticals. Harimurti et al. [130] developed a red dragon fruit extract encapsulated product in water-in-oil-in-water nanoemulsion as a delivery system ensuring antioxidant activities. In another study, the betalain microencapsulation added of potato succinylated starch increased the stability of stored yogurt [136].

Although the applications of nanoparticles for distribution, labelling, and heating have grown, their biological importance has been highlighted by the increase in their use and respect for the environment [137]. As a result, the manufacture of nanoparticles using plants or bio compounds has grown substantially. Tiny-sized gold nanoparticles with biostability are essential and could be employed in several biomedical applications. In a study, the authors produced gold nanoparticles (25.31 nm) using *H. polyrhizus* extract (pulp_seed oil extract), aiming to function as a reducing and stabilizing agent. Due to its substantial physiological stability and blood biocompatibility, green production of gold nanoparticles with red pitaya fruit extract is an alternative to chemical production and can be helpful for medical and biological applications. The authors used the nanoparticles in various biological assays, including anti-inflammatory, antioxidant, anti-diabetic, and anti-Alzheimer assays, and performed cytotoxic investigations. The anti-diabetic effects were shown by different concentrations of the nanoparticles (50 to 200 mg/mL), significantly inhibiting the alpha-amylase enzyme. The maximum anti-inflammatory action was obtained at 400 mg/mL with the inhibition of COX-1 (50.51 ± 1.32%) and COX-2 (58.74 ± 0.76%). The antioxidant effects were investigated at 100, 200, 300, 400, and 500 mg/mL of extract and pulp_seed oil. The antioxidant effects increased with the increase in concentrations. In the anti-Alzheimer investigation, Acetylcholinesterase and Butrylcholinearyltransferase were evaluated at different concentrations. Nanoparticles produced significant results at 400 mg/mL (inhibition of 69.11 ± 1.12% against Acetylcholinesterase and 64.78 ± 0.71% against butrylcholinearyltransferase). The inhibition actions were dose-dependent. In addition, nanoparticles exhibited a dose-dependent cytotoxic action against HepG2, HCT-116, and MCF-7 cell lines, showing as a viable anti-cancer therapy and an option for colon and breast cancer treatment. This study showed that these nanoparticles could be used as vectors for biomedical applications that may include medication and gene delivery or as a biosensor where they will be in contact with blood [75]. 

The fruit extract of *H. undatus* can also be used to produce nanoparticles, as shown by Rizvi et al. [138]. These authors worked on the green synthesis of iron oxide nanoparticles made from fruit extract of this species and observed efficient photocatalytic actions towards azo dyes. These results bring to light the possibility of using the genus *Hylocereus* to produce nanoparticles that can be used for pharmaceutical purposes or in the food industry. 

### 4.5. Waste Generated by Dragon Fruit Chain

Fruit and vegetable waste are severe environmental problems and are considered pollutants. They can significantly damage the aquatic and non-aquatic environment, resulting in severe inconvenience to animal and human organisms. For these reasons, the removal of this waste is highly urgent. The skin of the red pitaya is a residue from the pulp extraction or processing of the fruit’s juice and represents 33% of its weight. The volume of pitaya peel discarded worldwide is around 0.8 to 4 tons of fresh matter per hectare, making this fruit a source of waste that can be reused [80,139]. Bakar et al. [140] characterized the red pitaya skin and found high levels of dietary fiber (69.3%), most of which were insoluble but still with a good concentration of soluble fiber and considerable amounts of pectin (10.79%). Thus, applying pitaya peel flour can be a good alternative as a fiber or fat substitute in several products, enriching them nutritionally and sensorially [108]. Studies also suggest that pitaya peel pectin can be used as a functional and natural additive in low-viscosity beverages and foods [141,142].

Pitaya seeds possess an oil that is a mild laxative that reduces total and LDL-c levels in humans by inhibiting cholesterol absorption in the intestine. The oil has a high level of functional lipids and can be used as a new source of essential oil [143].

## 5. Conclusions

Studies show that pitaya has beneficial potential for human health, having antioxidant, anti-inflammatory, antilipemic, anti-diabetic, anti-bacterial, anti-fungal, and anti-cancer effects. The consumption of this fruit can act on oxidative stress and anti-inflammatory processes and control or reduce the occurrence of conditions such as diabetes, dyslipidemia, metabolic syndrome, cardiovascular diseases, and cancer. Pitaya has great economic importance due to its excellent nutritional value, and the possible use of peels, rich in betacyanins, can be applied as a natural dye. On the other hand, dietary fiber from fruit peels can be used to replace partial fat in products such as ice cream. The great potential for using pitaya in the food, cosmetics, and pharmaceutical industries can be demonstrated in the development of ecologically correct and active packaging, edible films, preparation of photoprotective products, natural additives for meat products, beer production, in addition to compounds that can act as natural cytotoxic agents in the therapeutic approach of various types of cancer.

Besides the importance of dragon fruit as a source of bioactive compounds, the bioavailability is low. The development of delivery systems such as gold nanoparticles with these compounds can be an alternative to reach target tissues.

The analysis of the studies included in this review shows us that dragon fruit has immense potential to improve numerous aspects of human health since it can reduce and treat risk factors for metabolic diseases in studies carried out with human beings and can present cytotoxicity in cancer cells. In addition, it has the potential to improve numerous technological aspects of food production and quality in terms of adding antioxidants and dietary fiber.

## Figures and Tables

**Table 1:** In vivo biological and pharmacological activities of various pitaya extracts and pure compounds.

Properties	Plant Part or Active Compounds	Species	Extraction	Experimental Model	Dose	Route of Administration	Methods of Analysis	Observations	References
Anxiolytic-like effects	Pulp and peel rich in maltotriose, quercetin-3-O-hexoside, and betalains bioactive compounds.	*H. polyrhizus*.	Ethanolic and aqueous extracts.	Zebrafish from both sexes.	0.1 mg/mL or 0.5 mg/mL or 1.0 mg/mL, 20 μL.	Dissolved in the fish’s water.	Anxiolytic activities and toxicity assays.	The extracts showed no toxicity in the fish model and exerted significant anxiolytic effects as the fish reduced their permanence in the clear zone of the experimentation area compared to controls.	[21]
Metabolic effects	Betalain-rich pitaya pulp.	*H. undatus.*	Aqueous extract.	Streptozotocin-induced diabetes in male Sprague-Dawley rats.	250 or 500 mg per kg body weight.	Intragastric gavage.	Pulse wave velocities, surgical procedures, and oxidative stress analyses.	The treated rats had reduced blood pressure, pulse wave velocities, and pulse pressures.	[22]
	Betalain-rich pitaya juice.	*H. polyrhizus*.	Aqueous juice.	High-carbohydrate, high-fat diet-induced metabolic syndrome male Wistar rats.	5% of red pitaya juice during the diet.	Oral by feeding.	Biochemical and physical tests, as well as histopathological assessments.	There was a significant reduction in the diastolic stiffness of the treated rats. Additionally, the treated rats presented reductions in the alkaline phosphatase and alanine transaminase serum concentrations.	[26]
	Pitaya’s juice.	*H. polyrhizus*.	Aqueous juice.	High-carbohydrate, high-fat diet-induced metabolic syndrome male Wistar rats.	5% of red pitaya juice during the diet.	Oral by feeding.	Biochemical and physical tests, as well as genetic assessments.	During the treatment, the omental and epididymal fat of the rats increased. The pitaya treatment reversed the rats’ metabolic changes by up-regulating the obesity-related Pomc and Insr genes in the liver tissues.	[27]
	Pitaya’s peel purified betacyanins.	*H. undatus.*	Purified betacyanins.	High-fat diet-fed male C57BL/6J mice.	Different concentrations of different purified betacyanins.	Oral by feeding.	Biochemical and histopathological analyses.	The purified betacyanin ameliorated the AT’s hypertrophy, liver steatosis, body glucose intolerance, and the body’s IR. In the liver, the purified betacyanins also augmented the genetic expression of lipid metabolism genes, such as the Acox1, Cpt1b, Cpt1a, Insig1, PPARγ, AdipoR2, and Insig2 and of FGF-21 genes. The purified betacyanins alleviated the liver’s FGF21 resistance, decreased the liver’s fatty acid biosynthesis, and elevated the liver’s fatty acid oxidation.	[28]
	Betacyanin-rich pitaya fruit.	*H. polyrhizus*.	Red pitaya’s fruit betacyanins.	Diet-induced obesity, liver steatosis, and insulin resistance C57BL/6J mice.	200 mg/kg.	Intragastric gavage.	Biochemical, sequencing, and histological analyses.	The fruit protected the mice from obesity and its related metabolic disorders. There were improvements in the inflammatory statuses of the treated rats, as well as their gut microbiota (there was a decrease in the ratio of *Firmicutes* and *Bacteroidetes* and an increase in the relative amount of *Akkermansia*).	[31]
	Pitaya juice rich in polyphenols and flavonoids bioactive compounds.	*H. undatus.*	Aqueous juice.	Steatosis diet-induced obese C57BL/6J mice.	-	Oral by drinking.	Biochemical and histopathological assessments.	There were improvements in the FGF-21 resistance and lipid metabolisms of the treated rats. Additionally, the juice protected the rats against hepatic steatosis and IR.	[32]
	Concentrated Pitaya Pulp with seeds	*H. polyrhizus*.	Pulp with seed	Hyperlipidemic female C57BL/6 mice	100, 200, and 400 mg/kg/day	Oral by feeding	Biochemical assessments.	There was an increase in the levels of HDL-c and a significant reduction in the levels of Total cholesterol, LDL, triglycerides, glycemia, AST, and ALT.	[23]
	Betacyanin-rich pitaya peel extract.	*H. polyrhizus*.	Methanolic extract.	Alcoholic-progressive liver disease ethanolic-diet C57BL/6 mice.	500 and 1000 mg/kg of body weight.	Intragastric gavage.	Biochemical and histopathological assessments.	The treated group presented diminished liver injury and improved liver lipid metabolism via decreases in the SREBP-1 and increases in AMPK and PPAR-α protein expressions. The extract also inhibited the Nrf2 and CYP2E1 expression, reduced endotoxin levels, and decreased TLR4, MyD88, TNF-α, and IL-1β expression in the treated rats’ liver.	[25]
	Concentrated Pitaya Pulp with seeds	*H. polyrhizus*.	Pulp with seed	Hyperglycemic Zebrafish	5–100% of pitaya pulp	Oral by feeding	Biochemical assessments.	There was a significant decrease in glycemia in all concentrations compared to placebo and with the use of metformin.	[24]
Gastrointestinal	Pitaya’s fruit extract.	*H. polyrhizus*.	Ethanolic extract.	Balb/c mice induced colitis by trinitrobenzene sulphonic.	1 g/kg.	Intraperitoneally.	Biochemical and histopathological analysis.	The extract exerted anti-inflammatory (decreases in the Ikb-a degradation and nuclear NF-kb protein levels) effects and prevented colitis development (reduced histological damage score) in the treated mice.	[29]
Wound-healing	Pitaya’s leaves, rind, fruit pulp, and flower extracts.	*H. undatus.*	Aqueous extract.	Streptozotocin diabetic Wistar rats	200 µL/wound at concentrations of 0.05%, 0.1%, 0.2%, 0.4% and 0.5% twice daily.	Topically.	Wound healing assays and DNA and protein estimation.	The use of the extract facilitated wound healing by enhancing tensile strength, hydroxyproline, DNA, total proteins, collagen content, and epithelization.	[30]

ALT—Alanine transaminase; AMP—adenosine monophosphate; AMPK—AMP-activated protein kinase; AST—aspartate aminotransferase; AT—adipose tissue; CYP2E1—cytochrome P450 2E1; FGF-21—fibroblast growth factor 21; Ikb-a—Ikb kinase alpha; Insr—insulin receptor gene; IL-1β—interleukin 1 beta; IR—insulin resistance; MyD88—myeloid differentiation primary response gene 88; NF-kb—nuclear factor kappa b; Nrf2—nuclear factor erythroid 2-related factor 2; PPAR-α—peroxisome proliferator-activated receptor; Pomc—proopiomelanocortin gene; SREBP-1—hepatic sterol regulatory element-binding protein 1c; TLR4—toll-like receptor 4; TNF-α—tumor factor necrosis alfa.

**Table 2:** Descriptive results of the biases found in the included animal studies following SYRCLE’S guidelines [15].

Properties	Study	Sequence Generation	Baseline Characteristics	Allocation Concealment	Random Housing	Binding (Intervention)	Random Outcome Assessment	Blinding (Outcome)	Incomplete Outcome Data	Selective Outcome Reporting	Other Sources of Bias
Anxiolytic-like effects	[21]	Yes	Yes	Yes	Yes	Unclear	Yes	Unclear	Yes	Yes	Yes
Metabolic effects	[22]	Yes	Yes	Yes	Unclear	Unclear	Unclear	Unclear	Yes	Yes	Yes
	[25]	Yes	Yes	Yes	Unclear	Unclear	Unclear	Yes	Yes	Yes	Yes
	[26]	Yes	Yes	Yes	Unclear	Unclear	Unclear	Unclear	Yes	Yes	Yes
	[27]	Yes	Yes	Yes	Unclear	Unclear	Unclear	Unclear	Yes	Yes	Yes
	[28]	Yes	Yes	Yes	Unclear	Unclear	Unclear	Unclear	Yes	Yes	Yes
	[31]	Yes	Yes	Yes	Unclear	Unclear	Unclear	Unclear	Yes	Yes	Yes
	[32]	Yes	Yes	Yes	Unclear	Unclear	Unclear	Unclear	Yes	Yes	Yes
	[23]	Yes	Unclear	Yes	Unclear	Unclear	Unclear	Unclear	Yes	Yes	Yes
	[23]	Yes	Yes	Yes	Unclear	Unclear	Unclear	Unclear	Yes	Yes	Yes
	[24]	Yes	Unclear	Yes	Unclear	Unclear	Unclear	Unclear	Yes	Yes	Yes
Gastrointestinal	[29]	Yes	Unclear	Yes	Unclear	Unclear	Unclear	Unclear	Yes	Yes	Yes
Wound-healing	[30]	Unclear	Yes	Unclear	Unclear	Unclear	Unclear	Unclear	No	Yes	No

**Table 4:** In vitro biological and pharmacological activities of various pitaya extracts and pure compounds.

Properties	Plant Part Used or Compounds	Species	Models	Type of Extract	Tested Concentrations	Methods	Observations	References
Anti-glycation	Freeze-dried powder rich in phenolic bioactive compounds.	*H. polyrhizus*.	Bovine serum albumin.	Ethanolic, hydro-ethanolic, methanolic, hydro-methanolic, acetone, aqueous, and petroleum ether extracts.	Concentrations range from 20 µg/mL to 100 µg/mL.	Glycation and aggregation of protein assays, carbohydrate cleaving enzyme inhibitory activity assessments, antioxidant studies, and determination of fructosamine inhibition.	Methanolic and acetone extracts were the most significant anti-glycation and antioxidant agents. The potential polyphenolic compounds associated with these effects were mainly 4-prenylresveratrol, vicenin, and luteolin.	[88]
	Leaves rich in triterpenes.	*H. undatus*.	Bovine serum albumin.	Chloroform extract.	0.5–2.0 mg·mL^−1^, 3 mmol·L^−1^, and 5 mmol·L^−1^.	Protein glycation and aggregation assessments.	The studied triterpenes could inhibit protein glycation at multiple stages, decreasing protein oxidation and protecting against diabetic-related complications.	[89]
Anti-diabetic	Bulb and peel.	*H. undatus*.	Dipeptidyl peptidase-IV enzyme.	Ethanolic and hydro- ethanolic extracts of pitaya’s fruit and ethanolic extract of pitaya’s peel.	10 mg/100 µL.	In vitro dipeptidyl peptidase-IV enzyme inhibition.	The hydro-ethanolic extract of pitaya’s fruit exerted 26.8 ± 0.55% of DPP-IV inhibition, the ethanolic extract of pitaya’s peel exerted 62.3 ± 0.63%, and the ethanolic extract of pitaya’s fruit exerted 84.2 ± 0.72%.	[90]
Anti-plasmodium	Peel.	*H. polyrhizus*.	*Plasmodium falciparum* 3D7 and W2 strains, HeLa cells, and SK-OV-3 cells.	Pigmented, n-hexane, dichloromethane, and ethyl acetate extracts.	-	Anti-plasmodium and cytotoxicity assays.	The dichloromethane extract demonstrated the most prominent anti-plasmodium activity at concentrations of 2.13 ± 0.42 µg/mL. The extracts showed cytotoxicity against the cancer cells at final concentrations of more than 1000 µg/mL.	[91]
Cytotoxicity	Pulp and peel rich in maltotriose, quercetin-3-O-hexoside, and betalains bioactive compounds.	*H. polyrhizus*.	Vero cell lines.	Ethanolic and aqueous extracts.	-	Cytotoxicity assays.	The toxicity against the cells ranged from 2.18 to 2.36 mg/mL for the peel and pulp.	[21]
	Peel.	*H. polyrhizus* and *H. undatus*.	PC3, Bcap-37, and MGC-803 cell lines.	Supercritical carbon dioxide extracts.	Maximum concentrations of 0.7 mg/mL.	Cytotoxicity assays.	All extracts demonstrated significant cytotoxicity against the cancer cell lines at concentrations ranging from 0.61 to 0.73 mg/mL.	[36]
Hepatoprotective	Peel rich in betacyanins.	*H. undatus*.	HepG2 cells.	Pressurized in the hot water extract.	Concentrations range from 20 μg/mL to 100 μg/mL.	Measurements of ROS and lipid accumulation, as well as biochemical tests.	The betacyanin-rich extracts could effectively exert antioxidant and hepatoprotective effects in the treated cells by controlling their lipid metabolisms and diminishing their TG contents. These compounds inhibited the liberation of the liver enzymes AST and ALT and probably regulated the mRNA expressions of the fatty acid synthase and carnitine palmitoyl transferase 1.	[92]
Anti-viral	Pulp rich in betacyanins.	*H. polyrhizus*.	Denv-2 strains and Vero cells.	Methanolic extracts.	Maximum concentrations at 4.346 mg·mL^−1^.	Cytotoxicity and anti-viral assays.	The extract was considered non-toxic for the cells at concentrations below 2.50 mg·mL^−1^. The most prominent anti-viral potential (95.0% virus inhibition) against the denv-2 was achieved at concentrations of 126.70 μg mL^−1^.	[93]
Immunomodulatory	Peel rich in the terpenoid lupeol.	*H. polyrhizus*.	Mice macrophages.	The compound was dry isolated.	100, 50, 25, 12.5 and 6.25 µg/mL.	Phagocytosis assessments and other macrophagic tests.	The terpenoid lupeol isolated from the mice was effectively associated with the increase of macrophage phagocytosis of latex beads, demonstrating potent immunomodulatory effects.	[94]
Bone effects	Fruit extract.	*H. polyrhizus*.	Bone marrow-derived mesenchymal stem cells.	Aqueous.	50, 100, 200, 300, and 400 µg/mL.	Cells osteogenic and proliferation assessments.	The fruit extract could promote the osteogenic differentiation and proliferation of the treated cells.	[95]

ALT—alanine aminotransferase; AST—aspartate aminotransferase; Bcap-37—human breast cancer cell line; Denv-2—dengue virus type 2; DPP-IV—dipeptidyl peptidase-IV; HeLa—cervical cancer cell line; HepG2—human hepatoma cell line; MGC-803—human gastric cancer cell line; mRNA—messenger ribonucleic acid; PC3—human prostate cancer cell line; ROS—reactive oxygen species; SK-OV-3—ovarian cancer cell line; TG—triglycerides.

**Table 5:** Effects of the genus *Hylocereus* in human studies.

Reference	Local	Model and Patients	Intervention	Outcomes	Adverse Effects
[20]	United Kingdom	Randomized, double-blind, placebo-controlled, cross-over trial with 19 healthy subjects (8 ♂, 11 ♀; 18–40 y)	Participants consumed 24 g of whole dragon fruit powder (33 mg of betalains) or a placebo daily/for 14 days. Blood pressure, Flow-mediated dilation (FMD), and arterial stiffness were evaluated (0, 1, 2, 3, and 4 h and 14 days after daily consumption).	Pitaya consumption significantly improved acute FMD at 2, 3, and 4 h post-consumption compared with placebo. This was also observed until 14 d. Pulse wave velocity was acutely significantly decreased at 3 h, and the augmentation index improved after 14 days compared with the placebo. No significant differences were observed for BP.	Not reported by patients.
[19]	Indonesia	Quantitative research type quasi-experiment (pre-test and post-test nonequivalent control group) with 32 students (4 ♂, 28 ♀; 21–26 y) who presented excess nutritional status.	Participants consumed 180 g of red pitaya/7 days.	The consumption of dragon fruit effectively reduced glycemia and blood pressure in subjects with excess nutritional status.	Not reported by the authors.
[17]	Malaysia	Randomized trial with 28 subjects (14 ♂, 14 ♀, 21 with type 2 diabetes; 20–55 y)	Participants were divided into 4 groups: 400 g of red pitaya/d, 600 g of red pitaya/d, negative control (diabetic subjects with normal diet), and the last group (healthy subjects with normal diet)/7 w. The study included 4 weeks of treatment and 2 weeks of wash-out.	After 4w: Group 1 showed a significant increase in HDL-c and a significant reduction of glycemia, LDL-c, and triglyceride. There was a significant augment of total cholesterol level on 7th w. No significant modifications were seen in group 2. No significant modifications were seen in weight and body fat in any group.	Not reported
[16]	Malaysia	Single-blinded cross-over trial with 36 Pre-diabetic participants	Pre-diabetic participants received 60, 80 or 100 g of spray pitaya (red) powder daily/4 week.	A significant decrease was seen in glycemia, total cholesterol, LDL-c, and triglycerides. A significant increase in HDL-c and total antioxidant status was observed.	Not reported
[18]	Malaysia	Single-blinded cross-over trial with 60 normocholesterolemic subjects	Participants were divided to receive 3, 4 or 5 sachets of spray pitaya (red) powder (20 g each) daily/4 weeks.	Significant reduction in glycemia, total cholesterol, LDL-c, and triglycerides. A significant increase in HDL-c and total antioxidant status.	Not reported by the participants.

HDL-c: High-density lipoprotein; LDL-c: Low-density lipoprotein.

**Table 6:** Description of the biases observed in the randomized clinical trials performed with the genus *Hylocereus*.

Study	Question Focus	Appropriate Randomization	Allocation Blinding	Double-Blind	Losses (<20%)	Prognostics or Demographic Characteristics	Outcomes	Intention to Treat Analysis	Sample Calculation	Adequate Follow-Up
[20]	Yes	Yes	Yes	Yes	Yes	Yes	Yes	NR	NR	Yes
[19]	Yes	No	No	No	NR	Yes	Yes	NR	NR	No
[17]	Yes	No	NR	NR	NR	Yes	Yes	No	No	Yes
[16]	Yes	No	Yes	No	NR	No	Yes	No	No	Yes
[18]	Yes	No	Yes	No	NR	No	Yes	No	No	Yes

NR—not reported.
